# Dietary patterns in Mexican preschool children are associated with stunting and overweight

**DOI:** 10.11606/s1518-8787.2021055002350

**Published:** 2021-08-06

**Authors:** Mario E. Flores, Marta Rivera-Pasquel, Nayeli Macías, Luisa María Sánchez-Zamorano, Sonia Rodríguez-Ramírez, Alejandra Contreras-Manzano, Edgar Denova-Gutiérrez

**Affiliations:** I Nutrition and Health Research Center National Institute of Public Health Cuernavaca México Nutrition and Health Research Center. National Institute of Public Health. Cuernavaca, México; II Population Research Center National Institute of Public Health Cuernavaca México Population Research Center. National Institute of Public Health. Cuernavaca, México

**Keywords:** Child, Preschool, Diet, Food, and Nutrition, Overweight, Growth Disorders, Nutrition Surveys

## Abstract

**OBJECTIVE:**

To evaluate the association between dietary patterns, stunting, and overweight among Mexican preschoolers.

**METHODS:**

This study was conducted with anthropometric (weight, height/length), sociodemographic (age, gender, education level of household head, socioeconomic status, country region and area, ethnicity, and beneficiary of social programs), and dietary data (Semiquantitative-food frequency questionnaire) on children aged from 1 to 4 years collected from the Mexican National Health and Nutrition Survey-2012. Dietary patterns were derived by principal components analysis. The association between dietary patterns, stunting, and overweight was assessed by prevalence ratios (PR), estimated by Poisson regression.

**RESULTS:**

In total, 1,112 preschoolers (mean age 3.06 years, SD = 1.08 years; 48.8% females) were included in the study; 11.9% of whom presented stunting, and 6.7% overweight. We identified four dietary patterns: Fruits and Vegetables [F&V], Western [W], Traditional [T], and Milk and Liquids [M&L]. Considering the lowest tertile of each dietary pattern as reference, the prevalence of stunting was 2.04 times higher [95%CI: 1.17–3.56] among children in the highest tertile of the “F&V” pattern. The prevalence of stunting was lower among children in the highest tertile of the “W” pattern [PR = 0.48; 95%CI: 0.27–0.85]. Overweight was negatively associated with the “F&V” dietary pattern [PR = 0.37; 95%CI: 0.16–0.85 for its highest tertile], and children whose consumption was mostly equivalent to the “T” pattern showed higher prevalence of stunting [PR = 1.74; 95%CI: 1.01–3.00].

**CONCLUSIONS:**

The prevalence of stunting and overweight in a nationwide sample of Mexican preschoolers was associated with dietary patterns.

## INTRODUCTION

Just as other low- and middle-income countries around the world, Mexico is undergoing an epidemiological and nutritional transition characterized by the double burden of malnutrition, changes in food consumption patterns, and increasing prevalence of diet-related chronic diseases^1 , 2^ . The Mexican National Nutrition Surveys (1988, 1999, 2006, and 2012) show that the prevalence of overweight among children under five years of age increased from 8.3% in 2006 to 9.7% in 2012^3^ . Other study indicates that the prevalence of overweight and obesity among school-age children increased from 18.6% in 1999 to 34.4% in 2012^4^ . The prevalence of stunting among Mexican children under the age of five is equal to 13.6%, whereas that of wasting is 1.6%^3^ . As a consequence of these conditions, obese children may experience immediate health consequences such as insulin resistance, hypertension, dyslipidemia, endocrine and gastrointestinal disorders, as well as psychological effects and social stigmatization^5^ . In turn, stunting can cause growth retardation, decreased learning capacity, impaired immune response, and the development of chronic illnesses in adulthood^6^ .

Dietary patterns, and consequently the quality and adequacy of food intake, are a major determinant of individuals’ nutritional status^7^ . As nutrients are not eaten in isolation, analyzing nutritional status based on isolated nutrients or food groups ignores the overall, complex effect of diet^8^ ; rather, studies approaching the relationship between nutrition, growth, and health in children should account for patterns of dietary intake^8^ .

A study conducted with preschoolers found those with dietary patterns rich in dairy, fruits, vegetables, protein, and carbohydrates to be less likely to present stunting^9^ . However, other studies reported conflicting association between dietary patterns and overweight and obesity among children^10 - 12^ . These findings indicate that the association between dietary patterns and nutritional status among preschoolers is still inconclusive, especially regarding obesity^10^ . Moreover, the literature on the topic lacks studies approaching Mexican preschool children.

The 2006 Mexican National Health and Nutrition Survey (ENSANUT) identified five dietary patterns among children aged between 5 and 11 years old, namely: a “Rural” pattern, rich in tortilla and legumes; a “Sweet cereal and corn dishes” pattern, high in sugary cereals, tortilla, and maize products; a “Diverse” pattern, including several food groups; a “Western” pattern, high in sweetened beverages, fried snacks, industrialized sweet bread, and sugary cereals; and a “Whole milk and sweets” pattern, high in whole milk and sweets. Children whose food intake consisted mostly that of the Western and Sweet cereal and corn dishes patterns presented an increased risk for overweight/obesity than those whose intake consisted mostly of the Rural pattern^13^ . However, the ENSANUT does not assess analogous relationships in these children’s younger counterparts.

Various factors influence eating behaviors during childhood and early childhood^4^ , such as geographical area, socioeconomic status, mother’s education level, and parental eating habits^4^ . In Brazil, children of mothers with higher levels of income and education consumed more fruits and vegetables, whereas those of mothers with lower levels of income and education were more prone to present a more traditional dietary pattern^14^ . Children at this life stage grow and develop rather quickly, acquiring eating behavior and food preferences. In countries bearing the double burden of malnutrition among preschoolers, identifying children’s eating patterns is important for determining interventions aimed at promoting dietary changes. Thus, this study sought to evaluate the association between dietary patterns, stunting, and overweight among Mexican children aged between 1 and 4 years old.

## METHODS

### Study Design and Participants

This study was conducted with data on 1,338 children aged from 1–4 years of age who participated in the Mexican National Health and Nutrition Survey-2012 (ENSANUT-2012). The ENSANUT-2012 was conducted from October 2011 to May 2012 with a probabilistic, multistage, stratified random sample, which provided representative data on 50,528 households located in urban and rural areas of three regions in Mexico at a national and state level (North, Center and Mexico City, South)^15^ . Dietary information was obtained by randomly applying a semi-quantitative-food-frequency questionnaire (SFFQ) to one out of every six subjects per age group. A detailed description of the sampling procedure was published in another study^16^ .

To assess the nutritional status of the population, Mexico has been conducting nationally representative nutrition surveys since 1988 with similar sampling framework and methodology organized by the same research team. Previous studies described these practices in detail^3 , 17^ .

Dietary data on 1,338 children were retrieved from the survey. After exclusion of non-plausible and missing dietary and anthropometric data (n = 126), agree we reached a final analytic sample of 1, 212.

### Ethics

This study was conducted according to the guidelines set by the Declaration of Helsinki. The survey protocol was approved by the Research, Ethics and Biosecurity Committee of the National Institute of Public Health (INSP, by its Spanish acronym), Mexico. Written informed consent was obtained from each subject’s parent or guardian for their participation in the study.

### Data Collection

#### Dietary information

Dietary information was collected with a previously validated^18^ 7-day SFFQ administered by trained health personnel. Data were recorded on laptop computers (Hewlett Packard 435) equipped with specific ENSANUT 2012 software (Visual Fox Pro program, v.7)^16^ . During the interview, guardians/caretakers were asked to recall, based on standard and home-made measurements, their children intake of each food item considering portion sizes, times-a-day, number of days of the week, and the number of portions.

According to a single 24-hour recall questionnaire from the 1999 Mexican National Nutrition Survey (ENN-99), foods included in the questionnaire represented 95% of the total dietary consumption of preschool-age children^16^ . However, a group of experts in nutrition from the Center for Nutrition and Health Research in the National Institute of Public Health in Mexico selected and added thirty-nine new commonly-consumed foods to the ENSANUT-2006 and ENSANUT-2012 questionnaires, classified according to fat, sugar and sodium content^16^ .

In this sense, the SFFQ included 123 food and beverage items, classified into 22 groups according to their characteristic (type, macronutrient, sugar, and fat content) to provide individuals’ dietary patterns. The research team responsible for assigning the food into groups reached a consensus regarding their classification: 1) Fruits; 2) Vegetables; 3) Meat, poultry and fish; 4) Stew; 5) Corn tortilla; 6) Beans; 7) Eggs; 8) Mexican *antojitos* (tacos, quesadillas); 9) Bread; 10) Cookies; 11) Milk; 12) Dairy; 13) Soups; 14) Oils and fat; 15) Fast food; 16) Snacks; 17) Sweets; 18) Desserts; 19) Ready to-eat-cereals; 20) Water; 21) Sugar-sweetened beverages; and 22) Sugar-free beverages ( Table 1 ).

**Table 1:** Food groups used in the dietary pattern analysis among Mexican preschoolers (ENSANUT-2012).

Food group	Example of foods
Fruits	Papaya, pineapple, strawberry, grapes, mango, peach, jicama, grapefruit, orange/tangerine, apple or pear, cantaloupe or watermelon, guava, banana, and dried fruits.
Vegetables	Carrot, cucumber, green beans, broccoli/cauliflower, nopal, squash, red tomato, dark green leaves (quelites, spinach, chard), green tomato, onion, chayote, zucchini, cabbage, lettuce, avocado, poblano pepper, corn, and canned vegetables (peas, carrots, mushrooms, and green beans).
Meat, poultry and fish	Beef, dried beef, pork, chicken, sausages, ham, tuna and sardine (tomato, water, or oil), fresh fish, dried fish (cod), seafood (shrimp).
Stew	Meat (chicken, beef) and vegetables prepared with other ingredients.
Corn tortilla	Corn tortilla.
Beans	Beans, lentils, chickpeas, yellow beans.
Eggs	Eggs.
Mexican Antojitos	Tacos, quesadilla, sopes, tostadas, tamal, pozole, atole.
Bread	White and whole wheat bread.
Cookies	Oreo, chocolate chips, and all kinds of sweet and salty cookies.
Milk	Whole milk.
	Low-fat milk.
Dairy	Yogurt and fresh cheese, mature cheese, Danone and Yakult’s products.
Soups	Vegetable soup, noodle soup, chicken soup, cream vegetable, and instant soups.
Oils and fat	Oils, margarine, butter, lard, mayonnaise, cream.
Fast food	Sandwich, pizza, hamburger, hot dog, pie.
Snacks	Potato chips, Doritos, salted pumpkin seeds, peanuts.
Sweets	Candies, donuts, cake, jellies, marshmallow, lollipops.
Desserts	Flan, Jell-O gelatin, pudding, ice-cream.
Ready to-eat-cereals	Branded cereals.
Water	Plain water – no sugar added.
Sugar-sweetened beverages	Sodas, Kool-aid, and sugar-sweetened fruit juices, flavored water, and tea.
Sugar-free beverages	Coffee and tea without sugar, diet soft drinks.

#### Identification of food patterns

Foods and food groups intake was calculated according to frequency, portion size (g, ml), and number of portions in every meal divided by 7 days of the week and expressed in grams (g) or milliliters (ml) per day. Then, using a food composition Table created by the National Institute of Public Health^19^ , the consumed amount (g or ml) of each of item was converted into percentage of total food intake per day, standardized, and expressed as *Z* -scores.

Dietary patterns were identified using principal components analysis and factor loadings. To keep factors uncorrelated and improve their interpretation, Varimax (orthogonal) rotation was applied. Four dietary patterns were obtained after evaluating eigenvalues (factors with an eigenvalue > 1.5), the proportion of variance explained, and interpretability. As suggested in a previous analysis^20^ , food groups with factor loading ≥ 0.20 were considered to significantly contribute to the pattern. Together, the four dietary patterns explained 34.3% of total variance – a value considered reasonable according to the literature^21^ . The factor score for each dietary pattern was calculated by adding food groups intake weighted by their factor loadings. Each participant received a factor score for each pattern.

#### Anthropometry

Data on body weight and height (length in children < 2 years old) were collected by trained personnel using standardized and international procedures^22^ and converted into *Z* -scores using the WHO/NCHS/CDC reference pattern^23^ . Based on their Z-scores, children were classified as stunted, [< -2 standard deviations (SD) for length/height-for-age] [adequate (≥ -1 and ≤ +1 weight-for length/height] or overweight [> 2 SD weight-for-height].

## Other Variables

### Socioedemographic data

A socioeconomic questionnaire was applied by trained and qualified personnel, collecting data on children’s age, gender (male, female), and socioeconomic status, as follows:

Education level of household head, classified as: Higher Education, High School, Up to Elementary School Household.

### Wealth Index (HWI)

A well-being indicator was created using principal components analysis (PCA), including housing conditions, flooring and roofing materials, home appliances and electronics (refrigerator, gas stove, washing machine, TV set, radio, video player, telephone, and computer), and number of rooms (not including bathrooms, kitchen, and hallways). The resulting standardized score was divided into tertiles, categorizing low, middle, and high *HWI* ^24^ .

### Region

The country was divided into three geographic regions: North (Baja California, Baja California Sur, Coahuila, Chihuahua, Durango, Nuevo León, Sonora, Tamaulipas); Central and Mexico City (Aguascalientes, Colima, Estado de México, Guanajuato, Jalisco, Michoacán, Morelos, Nayarit, Querétaro, San Luis Potosí, Sinaloa, Zacatecas, México City); and South (Campeche, Chiapas, Guerrero, Hidalgo, Oaxaca, Puebla, Quintana Roo, Tabasco, Tlaxcala, Veracruz, Yucatán).

### Area of residence

Based on the number of inhabitants, localities were classified as either rural (< 2500 inhabitants) or urban (≥ 2500 inhabitants).

### Ethnicity

When the household head reported being a speaker of indigenous language, the child was considered indigenous.

### Beneficiary of Social Welfare Programs

Individuals benefiting from food programs such as Oportunidades, LICONSA, and Programa Apoyo Alimentario (PAL) were considered as beneficiaries.

## Statistical Analysis

The general characteristics of the study participants underwent descriptive analysis. Continuous variables are presented as means and standard deviations, and categorical variables as percentages. To enable comparisons between extreme categories of dietary patterns, derived factors were categorized into tertiles and labeled based on the relative importance of the food groups in each pattern. Patterns scores were divided into tertiles, and general characteristics of interest were compared between the lowest and highest tertiles. Mean comparisons were performed using an analysis of variance (ANOVA). The association between dietary patterns, stunting, and overweight was assessed by prevalence ratios (PRs), determined by Poisson regression. Models were adjusted for dietary patterns and sociodemographic characteristics (region, HWI, and area), energy intake (kcal/day), energy residuals, and gender.

Using a regression model where nutrient intake is the dependent variable and energy intake the independent variable, energy residuals were calculated as a continuous variable and added to the model^25^ . All analyses were conducted considering the design effect and using the SVY module from the Stata software v.13.1^26^ .

## RESULTS

In total, 1,112 preschoolers were included in the study, 48.8% of whom were female. The mean age was 3.06 y among these, 11.9% presented with chronic undernutrition (stunting) and 6.7 % were overweight. Almost half of the children lived in the Center and Mexico City (47.3%), and 70.3% lived in urban areas. Around ten percent (9.6%) of the study sample was classified as of indigenous ethnicity, and 50.6 % were beneficiaries of social welfare programs ( Table 2 ).

**Table 2:** Characteristics of Mexican preschoolers (ENSANUT-2012).

Variable	Mean (SD)^a^	n sample^b^	%
***Age (y)***	3.06 (1.08)	1,112	---
**Weight (kg)**	14.1 (3.1)	1,112	---
**Height (cm)**	92.6 (9.5)	1,112	---
**Age (y)**			
1−2		539	48.3
3−4		573	51.7
**Gender**			
Female		587	48.8
Male		525	51.2
**Nutritional status** ^**c**^			
Adequate		864	80.6
Overweight		88	6.7
Stunting		149	11.9
**Household head education level** ^**d**^			
Higher education		245	25.5
High school		339	30.7
Up to elementary school		528	43.9
**HWI** ^**e**^			
High		378	39.4
Middle		363	32.9
Low		371	27.7
**Region**			
North		212	19.2
Center and Mexico City		461	47.3
South		439	33.5
**Area**			
Urban		671	70.3
Rural		441	29.7
**Indigenous ethnicity**			
No		978	90.4
Yes		134	9.6
**Beneficiary of social welfare programs** ^**f**^			
No		604	32.3
Yes		325	50.6
No information		183	17.1

^a^ Mean (Standard Deviation)

^b^ n = 1,112 observations

^c^ Adequate (≥ -1 and ≤ +1 W/H Z-score) Overweight (> +2 W/H Z-score); Stunting (< -2 H/A Z-score)

^d^ Household Head (mother, father/grandparents)

^e^ HWI, Household Wealth Index.

^f^ Any person in the household being beneficiary of one or more of the following social welfare programs of the Mexican government: LICONSA, DIF, PAL.

Table 3 shows the factor loadings for each food group of the four major dietary patterns, characterized as follows: 1) *Fruits and vegetables* (“F&V”: high in fruits, vegetables, dairy, and soups) *;* 2) *Western* (“W”: high in sweets, sugar-sweetened beverages, fast food, meat, poultry and fish, cookies, Mexican antojitos, snacks, and oils and fat); 3) *Traditional* (“T”: high in beans, corn tortillas, eggs, and Mexican antojitos); and 4) *Milk and liquids* (“M&L”: high in sugar-free-beverages, milk, and soups).

**Table 3:** Factor loading matrix for major dietary patterns of Mexican preschoolers (ENSANUT 2012)a.

Food groups	Factor Loading^b^			

	Fruits and vegetables	Western	Traditional	Milk/Liquids
Fruits	**0.3857**	_	_	_
Vegetables	**0.4241**	_	_	_
Meat, poultry, fish	_	**0.3232**	_	_
Stew	_	_	_	_
Corn Tortilla	_	_	**0.4541**	_
Beans	_	_	**0.5066**	_
Eggs	_	_	**0.2981**	_
Mexican antojitos	_	**0.2650**	**0.2003**	_
Bread	_	_	_	_
Cookies	_	**0.3115**	_	_
Milk	_	_	_	**0.2532**
Dairy	**0.2178**	_	_	_
Soups	**0.2273**	_	_	**0.2209**
Oils and fat	_	**0.2326**	_	_
Fast Food	_	**0.3248**	_	_
Snacks	_	**0.2455**	_	_
Sweets	_	**0.4178**	_	_
Desserts	_	_	_	_
Ready-to-eat cereals	_	_	_	_
Water	_	_	_	_
Sugar-sweetened beverages	_	**0.3919**	_	_
Sugar-free beverages	_	_	_	**0.2884**

^a^ Factor loadings ≥ 0.20 of each food group were considered as pertaining to a dietary pattern.

^b^ For simplicity, values ≥ 0.20 are presented.

Note: The four factors explained 34.3% of the total variance.

As shown in Table 4 , children included in the highest tertile of the “W” pattern were older than those from the highest tertiles of the “F&V” and “M&L” patterns. We found 60% of children from the highest tertile of the “W” pattern to be aged between 3 and 4 years – a higher proportion when compared to other patterns. Around 19% of children from the highest tertile of “F&V” and “T” patterns presented chronic undernutrition (stunting). Children from the highest tertile of the “W” dietary pattern presented the lowest prevalence of stunting (8.1%). Children from the highest tertile of “F&V” (2.9%) and “T” (4.3%) presented the lowest prevalence of overweight, whereas those from the third tertile of “W” showed the highest prevalence of overweight (8.2%). We verified an association between lower socioeconomic status and household head education level and the highest tertile of “F&V” and “T”. Children living in the Southern region presented the highest consumption of the “T” pattern, whereas those from Center and Mexico City presented the highest consumption of the “M&L”.

**Table 4:** Nutritional status and sociodemographic characteristics of Mexican preschoolers by tertile of dietary patterns (ENSANUT-2012).

Dietary patterns	Fruits and vegetables		Western		Traditional		Milk/Liquids	
				
Variables	T1	T3	T1	T3	T1	T3	T1	T3
**Age (m)**	35.5	36.3^a^	33.1	39.5^a^	35.2	37.9^a^	36.9	36.9
**Weight (** kg)	14.1	13.7	13.1	14.8^a^	14.2	14.0	14.1	14.2
**Height (** cm)	91.8	91.5^a^	89.0	94.7^a^	92.0	92.3	92.2	92.5
**Age, years (%)**								
**1** – **2**	50.5	50.6	59.3	39.7^a^	53.9	44.4	46.7	48.0
**3** – **4**	49.4	49.4	40.7	60.3^a^	46.1	55.6	53.3	52.0
**Gender %**								
Female	48.4	49.8	47.1	44.8	50.0	47.4^b^	49.6	55.0^c^
Male	51.6	50.1	52.9	55.2	50.0	52.6^b^	50.3	45.0^c^
**Nutritional status** ^**e**^ **(%)**								
Adequate	82.9	76.9^a,b^	76.1	83.7^a^	84.5	75.5^a^	79.3	78.5
Overweight	9.3	2.9^a,b,d^	4.8	8.2^a^	7.8	4.3	7.1	6.9
Stunting	7.5	18.8^a,b,d^	17.9	8.1^a^	6.9	19.5^a^	13.6	12.7
**Household head education level** ^**f**^ **(%)**								
Higher education	28.9	23.0^a^	19.9	28.4^b^	36.9	13.3^a,b,c^	20.7	30.0
High school	32.9	22.3^a^	27.7	31.1	29.1	31.6^a^	31.1	31.0
Up to elementary school	38.1	54.5^a^	52.4	40.5^b^	34.0	55.1^a^	48.2	39.0^d^
**HWI** ^**g**^ **(%)**								
High	44.5	31.6^a^	30.0	37.6^a,b^	54.1	19.2^a,b^	34.0	40.1^b,c,d^
Middle	32.6	34.6^a^	38.0	36.2^a,b^	32.4	35.1^a,c^	32.7	38.0^d^
Low	22.9	33.6^a^	32.1	26.1^a^	13.3	45.6^a,b,c^	33.2	22.0
**Region (%)**								
North	24.8	15.9^a^	12.9	27.4^a,b^	17.9	17.1^a,c^	23.0	16.0^d^
Center and Mexico City	47.4	44.6^a^	49.4	40.1^a,d^	58.0	37.0^a,d^	41.9	52.0^b,d^
South	27.7	39.4^a^	37.6	24.1^b,c^	24.1	45.9^a,c^	35.1	32.0^c^
**Area (%)**								
Urban	73.4	63.9^a^	64.1	70.7^a,b^	82.1	56.5^a,b,c^	68.3	70.0^c^
Rural	26.5	36.0^a^	35.9	29.3^a,b,c^	17.9	43.5^a,b,c^	31.6	30.0
**Indigenous Ethnicity (%)**								
No	95.1	78.9^a,b,c^	87.5	92.1	95.1	79.0^a,c^	89.4	90.3^b^
Yes	4.9	21.1^a,b,c^	12.5	7.9	4.9	21.1^a,c^	10.6	9.7^b^
**Beneficiary of social welfare programs** ^**h**^ **(%)**								
No	32.7	28.8	38.0	41.9^c^	42.8	32.6^b,c^	34.9	48.1^a^
Yes	50.2	48.7	62.0	58.1^c^	57.2	67.4^b,c^	65.1	51.9^a^

T1: Tertile1 of food pattern.

T3: Tertile 3 of food pattern.

^a^ P < 0.05 between tertiles of the same pattern (T1 *versus* T3).

^b^ P < 0.05 Inter-pattern differences (FruitsT3 *versus* T3 of the other dietary patterns).

^c^ P < 0.05 Inter-pattern differences (WesternT3 *versus* T3 of the other dietary patterns).

^d^ P < 0.05 Inter-pattern differences (TraditionalT3 *versus* T3 of the other dietary patterns).

^e^ Adequate (≥ -1 and ≤ +1 W/H Z-score); Overweight (> +2 BMI z-score); Stunting (< -2 H/A z-score).

^f^ Household Head (mother, father/grandparents).

^g^ HWI, Household Wealth Index.

^h^ Any person in the household beneficiary of one or more of the following social welfare programs of the Mexican government: LICONSA, DIF, PAL or any NGO.

We also found an association between the consumption of the “T” pattern and children living in rural areas, and between the consumption of “W” and “M&L” and children living in urban areas. Indigenous children were more prevalent within the top tertile of “F&V” and “T” patterns ( Table 4 ).

Table 5 shows the association between dietary patterns, stunting and overweight by tertile in prevalence ratio estimates (PRs) and 95% confidence intervals (95%CIs). For each outcome, two sets of models are presented – one unadjusted and the other adjusted for gender, age, HWI, energy intake, and energy residuals. We did not evaluate the association between dietary patterns with wasting due to the small sample size in this category (<1%). Children from the highest tertile of “F&V” were twice as likely to be stunted and presented a lower prevalence of overweight when compared to the lowest tertile of the same pattern. Conversely, the prevalence of stunting was lower among children from the top tertile of “W”. The prevalence of stunting was almost twofold among children from the highest tertile of “T” when compared with those from the lower tertile. We found no associations between the “M&L” dietary pattern and children’s nutritional status.

**Table 5:** Unadjusted and adjusted Prevalence Ratios (PR) and confidence intervals (95%CI) for the association between dietary patterns, stunting and overweight among Mexican preschoolers (ENSANUT-2012)

Dietary patterns^a^	Unadjusted						Adjusted					
	
	Stunting^b^			Overweight^b^			Stunting^b,c^			Overweight^b,c^		
			
	PR	95%CI	p	PR	95%CI	p	PR	95%CI	p	PR	95%CI	p
**Fruits and vegetables**												
Tertile 1	1.00			1.00			1.00			1.00		
Tertile 2	1.14	0.64–2.03	0.655	0.94	0.51–1.73	0.844	1.15	0.66–2.04	0.618	1.06	0.56–2.03	0.855
Tertile 3	2.36	1.45–3.85	0.001	0.32	0.15–0.70	0.004	2.04	1.17–3.56	0.012	0.37	0.16–0.85	0.019
**Western**												
Tertile 1	1.00			1.00			1.00			1.00		
Tertile 2	0.60	0.37–0.98	0.043	1.57	0.84–2.95	0.161	0.68	0.43–1.05	0.080	1.56	0.82–2.97	0.178
Tertile 3	0.41	0.25–0.67	0.001	1.97	1.03–3.77	0.040	0.48	0.27–0.85	0.012	1.77	0.95–3.30	0.073
**Traditional**												
Tertile 1	1.00			1.00			1.00			1.00		
Tertile 2	1.46	0.79–2.70	0.232	0.98	0.53–1.83	0.954	1.29	0.70–2.38	0.421	1.01	0.54–1.87	0.985
Tertile 3	2.60	1.63–4.13	0.001	0.59	0.28–1.25	0.169	1.74	1.01–3.00	0.047	0.61	0.27–1.39	0.241
**Milk/Liquids**												
Tertile 1	1.00			1.00			1.00			1.00		
Tertile 2	0.85	0.56–1.28	0.434	0.84	0.44–1.60	0.597	0.89	0.59–1.35	0.588	0.78	0.40–1.50	0.455
Tertile 3	1.05	0.69–1.60	0.804	0.92	0.48–1.74	0.796	1.11	0.75–1.65	0.602	0.90	0.46–1.78	0.762

^a^ Tertile 1 as reference

^b^ Stunting (< -2 H/A z-score); Overweight (> +2 BMI z-score)

^c^ Model adjusted by gender, age, Household Wealth Index, energy intake, and energy residuals.

## DISCUSSION

In a representative sample of Mexican preschoolers, we identified four dietary patterns associated with stunting and overweight. The prevalence of stunting was higher among children whose eating pattern was traditional or high in fruits and vegetables. In turn, the prevalence of stunting was lower among those with Western eating pattern. We also found children adopting the dietary pattern high in fruits and vegetables (F&V) to present lower chances of being overweight.

Comparing dietary patterns among different populations is a difficult task due to their cultural, geographical, and social characteristics. Yet, our results are consistent with those reported by other studies^9 , 27^ . In our study, children with eating habits mostly consistent with a “T” pattern (characterized by maize foods like tortillas and Mexican antojitos, as well as beans and eggs) were more likely to present stunting – an indicator of long-term malnutrition. This finding is in line with those reported by a study conducted with preschool children from a rural area of Kenya, where the risk for stunting was higher among children who consumed the traditional pattern – mainly represented by maize foods, fruits, and low dietary diversity^27^ .

Our results indicate that children with a “W” dietary pattern (high in sweets, sugar-sweetened beverages, fast foods, animal protein, cookies, snacks, and oils) are less likely to present stunting. This finding corroborates those reported by a study conducted with children from Tehran, which found a protective association between stunting and a carbohydrate-protein pattern, high in sweets and desserts, poultry, dairy, fruits, legumes, and visceral meats ^9^ . We also verified a positive association between stunting and the “F&V” dietary pattern, in contrast with a study conducted with children aged from 6–23 months, which verified an association between attained linear growth (height-for-age- *z* -score) and low fruits and vegetables consumption^28^ . Another study conducted in Ethiopia verified an inverse association between stunting and a high intake of dairy products, vegetables, and fruits^29^ , which may be explained by the presence of animal protein (dairy).

In our study, both “F&V” and “T” eating patterns were associated with less favorable socioeconomic conditions, lower household head education level, and indigenous ethnicity (in the “T” pattern) – sociodemographic data previously associated with stunting^30^ . In this sense, the association between stunting and “F&V” and “T” patterns found in our study may be related to the limited intake of animal protein and other important nutrients for growth, such as Zn, Calcium, vitamin D, vitamin B12, and essential fatty acids. However, we cannot discard the possible influence of high morbidity rates among children of low socioeconomic status, mostly from diarrhea and upper respiratory infections. The “F&V” dietary pattern identified in our study cannot be considered a healthy pattern, for it lacks foods with essential nutrients for the growth and development of toddlers and preschoolers. Similar to other studies, our results indicate a negative association between the “W” pattern (rich in protein and other essential nutrients) and stunting, demonstrating that adequate protein intake is required for normal growth^27^ .

A European cohort study with preschool and school-age children reported at least two dietary patterns similar to those identified in our study, namely “F&V” and “W”^31^ . The authors found a positive association between adiposity and a dietary pattern termed “Junk or processed food,” mainly characterized by higher consumption of white bread, low-fiber breakfast cereals, French fries, whole milk, chocolate/confectionery, and crisps – similar to our “W” pattern^31^

In another study conducted with 403 Brazilian children aged from 4 to 7 years^32^ , an “Unhealthy” dietary pattern (fast food, snacks, sweets, sugary beverages, biscuits) was also positively associated with adiposity. However, differently from the children in our study whose mean age was 3 years, the mean age of the children in this study was 6 years^32^

We found no association between overweight and the “W” pattern, which may be explained by the low prevalence of overweight among these preschool-age children. This assumption is supported by national surveys that have shown that the prevalence of overweight increases with age [26.9% (1999) and 34.8% (2006) in school-age children]. Another hypothesis related to the young age of our study sample (1–4y) is that their exposure to “W” pattern eating habits was still not long enough to enable a considerable development of overweight.

A recent Mexican survey that evaluated dietary patterns^4^ verified an “Industrialized” eating pattern among school-age children, characterized by high consumption of snacks, fast food, sweets, sugar, and sweetened beverages – roughly similar to our “W” pattern. In our study, the “W” dietary pattern was positively associated with age, suggesting that a “Westernized”-like eating habit begins in infancy and is more markedly expressed during childhood^4^ .

We verified a positive association between the “W” pattern and a higher socioeconomic status and household head education level. However, this finding is contradicted by a study conducted with a different population of children^31^ where the “W” dietary pattern was associated with lower socioeconomic status, and education level was positively associated with a healthier diet.

Two recent reviews suggest that the association between dietary patterns and socio-demographic factors in childhood^32 , 33^ varies according to the wealth and development of the studied population. One of these, including seven studies conducted in developed countries (U.S, Europe, Japan and Australia)^33^ , found that higher levels of maternal education and income, as well as their age range, were associated with lower consumption of an “Unhealthy/Western” and higher consumption of a “Healthy/Prudent” pattern in children < 24 months. Four of these studies also verified a positive association between increased consumption of an “Unhealthy/Western” dietary pattern and lower levels of maternal income and education^33^ .

In a review conducted by deFragas-Hinning et al. including 40 published articles ^34^ , the authors verified an association between higher levels of education and income and increased consumption of a “Healthy” pattern among children aged < 24 months from highly-developed societies, as well as a consequent lower consumption of an “Unhealthy” pattern. However, a different study^34^ identified a positive association between an “Unhealthy/Western” dietary pattern and higher levels of income and education in middle and to some extent low-income societies, which we believe to be the case of Mexico.

Our study has some limitations. First, the cross-sectional design prevented us from inferring causality. Second, the semi-quantitative-food-frequency questionnaire (SFFQ) used for collecting dietary data may underestimate preschoolers’ food intake. However, previous studies reported that around 40–50% of score-based dietary patterns obtained with this instrument is accurately classified, as well as data obtained with a 5-day weighted food record^35^ . Although validated in a sample of Mexican adults^10^ , the instrument used for data collection on our study was not validated for use and reporting of preschool children’s food intake, but the dietary data on preschoolers obtained by SFFQ was comparable with those obtained by a 24-hour recall in a nationwide sample of preschool and school-age children^36^ .

Regarding its strengths, our study was conducted with a large, nationally representative sample. To adjust the association models, avoid confusion, and give a broad context of food patterns among preschool-age children in Mexico, we included socioeconomic and demographic characteristics such as region, place of residence, and ethnicity. Moreover, techniques and interviews on eating habits and anthropometric measures were carefully standardized.

## CONCLUSIONS

In a nationwide representative sample of Mexican preschoolers, we identified four dietary patterns associated with stunting and overweight. The prevalence of stunting was higher among children whose dietary pattern was mostly consistent with “Fruits and vegetables” and “Traditional”, but lower among those with higher adherence to “Western” pattern. None of the four dietary patterns identified were considered suitable for the adequate nutritional status of Mexican preschoolers, indicating the need for preventing childhood stunting by promoting a healthy diet that includes local foods rich in protein and essential nutrients. Our results provide further understanding on the dietary patterns of children aged between 1 and 4 years, and can be used to promote health education and campaigns aimed at early healthy eating to improve child growth and health among Mexican children.
